# A brief report on making meaning of FoMO: post-Covid mental health and wellbeing in Minority Serving Institute

**DOI:** 10.1007/s44192-025-00240-3

**Published:** 2025-07-03

**Authors:** Peri Yuksel, Wei Zhang

**Affiliations:** Department of Psychology, New Jersey City University, 2039 John F. Kennedy Boulevard, Jersey City, NJ 07305 USA

**Keywords:** FoMO and COVID-19 aftermath life perception, Anxiety, Post-pandemic lifestyle aspects, Social media usage in diverse adults

## Abstract

Understanding the dynamics of Fear of Missing Out (FoMO) remains critical in the post-pandemic era, as societies gain access to myriad forms of being connected. This study examined online and offline behaviors, mental health, FoMO, and Post-Pandemic Lifestyle Changes Inventory in 129 anonymous online users aged 18–64 (M = 27.43, SD = 10.941, 14.7% male vs. 85.3% female). FoMO showed a significant positive correlation with anxiety (r = 0.384, *p* < 0.001) and depression (r = 0.345, *p* < 0.001), while no significant links were found between FoMO and sample characteristics (i.e., education level, gender, sexual orientation, place of birth, ethnicity, online, or offline behavior). Higher FoMO scores correlated with less perceived improvement in mental, emotional, social, professional, and financial domains post-Covid-19, indicating a potential barrier to overall life satisfaction. Findings highlight the need for further research into the psychosocial implications and complexities of media use in the context of pandemic-driven lifestyle changes.

## Background

The Internet has revolutionized communication, fostering unprecedented connectivity through social networks [3]. The COVID-19 pandemic underscored the essential role of digital platforms in daily life, emphasizing their impact on social dynamics and community building [14, 15]. Despite their benefits in fostering social capital and alleviating loneliness, social media platforms raise concerns about their impact on mental health, particularly through phenomena like Fear of Missing Out (FoMO) [13, 18, 19]. Fear of Missing Out (FoMO) has become a well-established psychological phenomenon, recognized for its influence on online behavior and mental health outcomes [22]. It is theorized to stem from unmet social relatedness needs, which are further exacerbated by the pervasive nature of social comparisons on social media [7, 16]. As a prominent psychosocial construct, FoMO is characterized by a persistent concern that others are engaging in rewarding experiences in one’s absence [18, 19]. Empirical research has linked FoMO to problematic social media use, heightened anxiety, depression, and diminished well-being [6, 10]. Its impact on digital behavior is particularly evident in compulsive engagement with social media, passive scrolling, and excessive checking, all of which have been associated with increased psychological distress [21].

### Theoretical background

The theoretical underpinnings of FoMO can be explained through Self-Determination Theory (SDT) [7], which suggests that deficits in autonomy, competence, and relatedness increase individuals' susceptibility to FoMO-driven social comparison. When these fundamental psychological needs remain unmet, individuals are more likely to seek validation and fulfillment through digital interactions, often leading to heightened engagement with social media. Additionally, Social Comparison Theory [12] provides further insight into FoMO’s psychological mechanisms, emphasizing that digital environments facilitate frequent upward social comparisons, reinforcing self-evaluative concerns and dissatisfaction [21]. The constant exposure to curated online portrayals of others' lives intensifies perceptions of social exclusion and inadequacy, which may, in turn, contribute to mental health challenges. During the COVID-19 pandemic, increased reliance on digital communication intensified FoMO experiences, exacerbating psychological distress across multiple domains [4]. Longitudinal studies indicate that FoMO is a significant predictor of smartphone addiction, reduced emotional regulation, and heightened stress levels over time [5]. Given these findings, it is crucial to examine how FoMO influences post-pandemic life satisfaction and mental health, particularly as individuals navigate evolving social norms and digital behaviors in the aftermath of global disruptions.

### Aim

This study sought to expand prior research by exploring the relationship between FoMO, anxiety, depression, and perceived post-pandemic lifestyle changes. Given that this is a correlational study, causal claims are not made. Instead, the study tests the following hypotheses:

1. Higher FoMO scores will be positively correlated with anxiety and depression.
2. FoMO will be negatively associated with perceived post-pandemic improvements in emotional, social, and professional life domains.
3. Demographic variables (e.g., gender, ethnicity, education level) will not significantly predict FoMO, aligning with prior research suggesting FoMO is a universal rather than demographically bound phenomenon.

As society navigates heightened digital connectedness in the post-COVID era, understanding the psychological mechanisms underlying FoMO is essential for promoting mental well-being, fostering healthier online behaviors, and adapting to evolving societal norms.

## Methods

### Participants

Participants (N = 129) were diverse across ethnicity (38% Latinx, 19.4% Black/African American, 13.2% European, 6.2% Asian), education (69% undergraduate, 17.8% graduate), sexual orientation (68% heterosexual) and birthplace (29.5%, non-US born), and age (M = 27.43, Median = 23, SD = 10.94). To determine the appropriate sample size for this study, a power analysis was conducted using G*Power 3.1 [11]. Assuming a medium effect size (*f*^*2*^ = 0.15), *α* = 0.05, and power (1 − *β*) = 0.80, a minimum sample size of 89 participants was required for detecting significant associations in regression analyses. The final sample of 129 participants exceeded this threshold, ensuring adequate statistical power.

### Design

The research protocol was approved by the Institutional Review Board of New Jersey City University in accordance with the ethical standards as laid down in the 1964 Declaration of Helsinki and its later amendments or comparable ethical standards.

### Recruitment

Participants were recruited through university-wide digital communication platforms and social media groups of a Minority Serving Institution in an urban setting. Invitations were distributed via student forums, departmental newsletters, faculty announcements, and email lists, reaching an estimated 7000 individuals. Participation was voluntary and anonymous, with no financial incentives provided. Out of approximately 7000 individuals invited, 129 completed the survey, yielding a response rate of approximately 1.84%. Given the non-random convenience sampling, potential self-selection bias is acknowledged as a limitation, as individuals with a pre-existing interest in mental health or social media behaviors may have been more likely to participate. Future studies should consider employing randomized recruitment strategies or stratified sampling methods to enhance generalizability.

### Measures

Informed Consent was obtained from all the participants involved in the study to participate in an anonymous 20-min online Qualtrics survey that assessed post-pandemic lifestyle habits and attitudes towards online and offline behavior, mental health, and FoMO level.

1. Fear of Missing Out (FoMO) Scale [18]: This 17-item scale assessed anxiety and fear related to missing out on social experiences due to digital disconnection. The items are scored on a scale of 1 (not at all true of me) to 5 (extremely true of me), items include statements such as “I feel obsessed when I miss events/ opportunities,” “I get curious when I do not keep informed about the conversations between my friends,” and “I feel outcast from my social groups when I decline their invitation. The reliability of the FoMO scale in the current study was α = 0.924 (Cronbach’s alpha).
2. Patient Health Questionnaire (PHQ-4) [17]: This 4-item questionnaire measured anxiety and depressive symptoms on a scale of 0–3 (not at all, several days, more than half the days, nearly every day). It asks questions about how often within the last two weeks participants, for example experienced “feeling nervous, anxious or on edge” or “Feeling down, depressed or hopeless”. The reliability of the PHQ in the current study was α = 0.879.
3. Post-Pandemic Lifestyle Change Inventory (PPLCI) [23]: Developed specifically for this study, this scale measured perceived changes in lifestyle habits following the COVID-19 pandemic across various domains, i.e., sleeping habits, eating habits, mental health, social life, emotional aspects, financial situation, professional development, and physical activity, with scores from 1 (disagree strongly) to 5 (agree strongly). The scale was theoretically informed by Self-Determination Theory [7], which emphasizes how autonomy, competence, and relatedness influence individuals' psychological adaptation to life changes. Given the significant disruptions to daily life due to the pandemic, we constructed the PPLCI to assess perceived lifestyle adjustments across multiple domains, including mental health, emotional well-being, social life, professional development, and financial stability. A literature review on post-pandemic behavioral adaptations (e.g., [4]) guided the initial pool of items. Higher scores indicated more positive perceived changes. PPLCI demonstrated strong internal consistency (α = 0.847).

### Data analysis

Data analysis involved correlational analyses, ANOVAs, and t-tests to explore the relationships between FoMO and participant characteristics. Regression analyses adjusted for confounders (such as age, gender, sexual orientation, ethnicity, education, and birthplace) explored FoMO's effects on mental health and lifestyle post-COVID.

## Results

### FoMO and individual characteristics

FoMO was marginally correlated with age (r = − 0.166, *p* = 0.059), with younger participants showing higher FoMO levels. An independent samples t-test showed no significant gender differences in FoMO (t = − 0.641, *p* = 0.522), nor differences based on place of birth (t = − 1.299, *p* = 0.196). A one-way ANOVA revealed no significant differences in FoMO across sexual orientation groups (F = 2.08, *p* = 0.106) or ethnic groups (F = 1.461, *p* = 0.197). These results suggest that while age is inversely related to FoMO, other demographic factors do not show significant associations (all *p*s > 0.1).

### FoMO and mental health

FoMO was significantly correlated with anxiety (r = 0.384, *p* < 0.001, 95% CI [0.226, 0.522]) and depression *(r* = 0.345, *p* < 0.001, 95% CI [0.183, 0.489]), as confirmed by GLM analyses adjusting for confounders (both *p*s < 0.001, Table 1). FoMO did not correlate with the total count of diagnosed psychological disorders (r = 0.108, *p* = 0.222, 95%CI [− 0.066, 0.276]). This measure, derived from participants' self-reported previous diagnoses, suggests FoMO’s specific association with current anxiety and depression.

**Table 1:** GLM results: predictive effects of FoMO on mental health in participants (N = 129)

Anxiety					Depression					Psychological disorders				
Source	df	Mean square	F	*p*	Source	df	Mean square	F	*p*	Source	df	Mean square	F	*p*
Model	18	160.048	52.533	0.000	Model	18	121.51	42.461	0.000	Model	18	14.34	6.389	< 0.001
Gender	1	0.600	0.197	0.658	Gender	1	0.16	0.056	0.814	Gender	1	0.133	0.059	0.808
Sexual orientation	3	3.786	1.243	0.298	Sexual orientation	3	3.028	1.058	0.370	Sexual orientation	3	3.083	1.374	0.255
Ethnicity	6	6.037	1.982	0.074	Ethnicity	6	2.253	0.787	0.582	Ethnicity	6	5.405	2.408	0.032
Education	4	8.915	2.926	0.024	Education	4	5.512	1.926	0.111	Education	4	4.684	2.087	0.087
Birthplace	1	2.885	0.947	0.333	Birthplace	1	15.729	5.496	0.021	Birthplace	1	2.872	1.28	0.260
Age	1	10.649	3.495	0.064	Age	1	5.333	1.864	0.175	Age	1	1.452	0.647	0.423
**FoMO**	**1**	**63.938**	**20.987**	**< 0.001**	**FoMO**	**1**	**36.776**	**12.851**	**< 0.001**	**FoMO**	**1**	**4.142**	**1.845**	**0.177**
a R Squared =.896 (Adjusted R Squared =.879)					a R Squared =.874 (Adjusted R Squared =.854)					a R Squared =.511 (Adjusted R Squared =.431)				

### FoMO and post-Covid 19 lifestyle change

FoMO did not correlate with perceived improvements in eating habits (*p* = 0.343) but marginally affected sleeping (*p* = 0.069) and physical health (*p* = 0.088). Higher FoMO scores significantly correlated with lower perceived improvements in mental health (*p* = 0.001), emotional life (*p* = 0.001), social life (*p* = 0.004), professional development (*p* = 0.011), and financial situations (*p* = 0.024, Table 2). Regression analyses adjusting for confounders confirmed these associations.

**Table 2:** Correlations between FoMO and post-Covid 19 life aspects in participants (N = 129)

	FoMO	Eating habits	Sleeping habits	physical health	Mental health	Emotional life	Social life	Professional development	Financial situation
FoMO	1	− 0.084	**− 0.161**^**+**^	**− 0.151**^**+**^	− 0.278**	− 0.298**	− 0.253**	− 0.222*	− 0.199*
Eating habits		1	0.317**	0.452**	0.330**	0.273**	0.205*	0.247**	0.315**
Sleeping habits			1	0.365**	0.331**	0.448**	0.276**	0.263**	0.410**
Physical health				1	0.340**	0.391**	0.367**	0.137	0.147
Mental health					1	0.804**	0.518**	0.506**	0.417**
Emotional life						1	0.554**	0.503**	0.413**
Social life							1	0.456**	0.341**
Professional development								1	0.416**
Financial situation									1

**p < 0.01, *p < 0.05, ^+^*p* < 0.1

## Discussion

This study aimed to explore how FoMO interacts with demographic factors, psychological variables, and post-COVID life aspects among adult online users. Findings revealed insights into mental health implications and strategies to mitigate anxiety and depression post-pandemic.

### FoMO and demographic variables

Research has varied on FoMO's association with demographics such as age, gender, education level, and ethnicity [2, 8, 9]. Our correlation analysis found a slight inverse relationship between FoMO and age (*p* = 0.059), consistent with previous findings indicating younger individuals tend to experience higher FoMO, underscoring FoMO's complex interaction with demographics [18, 19, 22].

While the current study found no significant associations between FoMO and demographic factors (gender, ethnicity, education level, sexual orientation) through correlation, t-tests, and ANOVA analyses (all *p*s > 0.1), prior literature has suggested otherwise. For example, Barry and Wong [2] and Dou et al. [8] found that younger adults and individuals from certain cultural backgrounds experience heightened FoMO due to social comparison tendencies. One possible explanation for the discrepancy in our findings could be the demographic composition of our sample, which consists predominantly of college students at a Minority Serving Institute, where shared digital experiences may overshadow individual demographic differences. To explore potential moderation effects, we conducted post-hoc subgroup analyses examining whether age, gender, or ethnicity moderated the relationship between FoMO and mental health outcomes. No significant interaction effects were found (*p* > 0.1), suggesting that FoMO’s psychological impact may be more universally experienced across diverse groups in this sample. Future studies should employ larger, more representative samples and longitudinal designs to assess whether these demographic variables play a moderating role over time.

FoMO has been consistently linked to anxiety and depression [10, 18, 19]. Our regression analysis further confirmed this relationship, with significant associations persisting even when considering gender, sexual orientation, education, and ethnicity (*p* < 0.001 for both anxiety and depression), suggesting that FoMO independently contributes to mental health outcomes. FoMO has been consistently associated with heightened anxiety and depression [10, 18, 19]. Research suggests that this relationship may be partially explained by increased social comparison and perceived inadequacy, as individuals with higher FoMO tend to engage more frequently in upward social comparisons, leading to greater dissatisfaction and psychological distress [21]. While our study did not directly measure these intervening variables, the observed correlation between FoMO and psychological distress aligns with established theoretical frameworks, such as Social Comparison Theory [12] and Self-Determination Theory [7], which explain how FoMO-driven digital engagement may contribute to negative self-perceptions and emotional well-being challenges. These findings underscore the importance of further empirical research to examine potential mediating mechanisms and reaffirm FoMO’s broader implications for mental health.

### FoMO and lifestyle changes post-COVID 19

The COVID-19 pandemic intensified feelings of isolation, potentially exacerbating FoMO among digital platform users [14]. A notable finding was that higher FoMO was significantly associated with lower perceived improvements in mental, emotional, social, professional, and financial well-being, yet showed no significant association with physical health, as supported by both correlation and regression analyses. This pattern suggests that FoMO is primarily a social-cognitive phenomenon, driven by perceived social exclusion and digital comparison [18, 19], rather than an objective indicator of health behaviors. One possible explanation is that physical health behaviors (e.g., diet, exercise, sleep) are influenced more by external constraints (e.g., work schedules, physical environment) than by social comparison processes. In contrast, mental and emotional well-being are highly sensitive to perceived social inadequacy, which FoMO amplifies through constant exposure to curated online lifestyles [10].

Future studies should examine whether specific coping mechanisms, such as mindfulness training, cognitive reframing, or digital detox interventions, can mitigate FoMO’s detrimental impact on mental health. Additionally, investigating differences in coping strategies between high- and low-FoMO individuals could offer insights into resilience-building in the post-pandemic digital era. Targeted interventions need to address FoMO-related distress following post-pandemic norms of communication and interaction [6].

### Practical implications: potential FoMO coping strategy

While the psychological impact of FoMO on mental health is well-documented [10, 18, 19], less is known about effective coping mechanisms and intervention strategies to mitigate its effects. Research suggests that mindfulness-based approaches, cognitive restructuring, and digital detox interventions may help individuals regulate social comparison tendencies and reduce FoMO-related distress [1, 20]. For instance, mindfulness training encourages present-moment awareness, potentially counteracting the anticipatory anxiety of missing out on social experiences. Additionally, self-regulation strategies, such as limiting passive social media use, fostering real-life social connections, and setting intentional online consumption habits, have shown promise in reducing FoMO’s impact on anxiety and depressive symptoms [6]. Future research should examine longitudinal interventions, exploring whether structured programs (e.g., social media literacy training, therapy modules targeting FoMO) can effectively mitigate its psychological toll. Integrating these coping strategies into mental health awareness programs on college campuses and in workplaces could provide practical tools to improve digital well-being and overall life satisfaction in a post-pandemic society.

### Theoretical integration

The findings align with broader psychosocial theories, particularly Social Comparison Theory [12] and Self-Determination Theory [7], which provide insight into the mechanisms through which FoMO influences mental health and lifestyle perceptions. From a Social Comparison Theory perspective, FoMO is fueled by constant exposure to curated online portrayals of others’ lives, leading to upward social comparisons and heightened self-evaluative concerns [21]. Individuals with higher FoMO may engage in frequent passive social media consumption, leading to increased dissatisfaction with their own experiences, particularly in the domains of emotional well-being, social interactions, and professional life—as supported by this study’s findings.

In contrast, Self-Determination Theory (SDT) posits that FoMO may arise from unmet psychological needs for autonomy, competence, and relatedness [7]. The significant correlations observed between FoMO and negative mental health outcomes (anxiety, depression) suggest that individuals experiencing high FoMO may struggle with feelings of social disconnection and lack of self-determined agency in their digital engagement. Post-pandemic shifts in social behaviors may have exacerbated these concerns, reinforcing the need for interventions that promote intrinsic motivation and offline social fulfillment to counteract the adverse effects of digital comparison-driven FoMO. Future research should explore individual differences in resilience to FoMO, particularly through the lens of these psychosocial theories, to identify protective psychological factors that may buffer against FoMO’s negative impact on mental health and life satisfaction.

## Limitation and future direction

The authors acknowledge the small sample size in this correlational study as a limitation, hence results should be interpreted with caution. However, this brief communication encourages future research to explore lifestyle habits post-COVID in relation to screen time including diverse adult populations. Understanding FoMO is critical, particularly for recognizing its impact on young adults susceptible to social comparison and peer pressure [1]. Future research should prioritize longitudinal studies to monitor the frequency, purpose, and nature of social media usage, examining their influence on both online and offline lifestyle habits, including sleep patterns and dietary behaviors, to gain a deeper understanding of FoMO. Moreover, exploring the potential of popular online platforms and support groups in mitigating FoMO among diverse internet users, including those with existing psychopathology and digital natives, could significantly enhance overall quality of life in an era characterized by constant real-time digital updates facilitating social comparison.

## Data Availability

The data and supplementary materials supporting the findings of this study are available and can be requested from the corresponding author.

## Appendix

See Tables 3, 4, 5, 6, 7, 8, 9 and 10.

**Table 3:** FoMO's impact on perceived improvement in eating habits post-COVID: regression results

Predictor	Categories	Estimate	SE	Wald	*p*	95% confidence interval	
						Lower bound	Upper bound
**FOMO**		**− 0.013**	**0.013**	**0.985**	**0.321**	**− 0.038**	**0.012**
Age		− 0.013	0.021	0.373	0.541	− 0.055	0.029
Gender	Male	− 0.016	0.489	0.001	0.974	− 0.974	0.942
	Female	0^a^					
Sexual orientation	Heterosexual	0.749	0.543	1.900	0.168	− 0.316	1.813
	Homosexual	− 0.286	0.950	0.091	0.763	− 2.147	1.575
	Bi-sexual	0.323	0.644	0.252	0.616	− 0.940	1.586
	Other	0^a^					
Ethnicity	Asian	− 0.072	0.850	0.007	0.932	− 1.738	1.593
	Black/African American	0.445	0.654	0.463	0.496	− 0.837	1.728
	European	− 0.632	0.735	0.740	0.390	− 2.073	0.808
	Latinx	− 0.149	0.601	0.062	0.804	− 1.327	1.029
	Middle Eastern	− 0.606	1.001	0.366	0.545	− 2.569	1.357
	Mixed	− 0.123	0.763	0.026	0.872	− 1.618	1.372
	Other	0^a^					
Education	High school	− 3.586	1.232	8.476	0.004	− 6.001	− 1.172
	Currently undergraduate student	− 1.442	0.857	2.832	0.092	− 3.121	0.237
	Currently graduate student	− 0.469	0.861	0.296	0.586	− 2.156	1.219
	Master's Degree	0.617	0.941	0.430	0.512	− 1.227	2.461
	Beyond Masters/PhD/Professional Degree	0^a^					
Birthplace	Outside of USA	− 0.076	0.379	0.040	0.841	− 0.819	0.666
	USA	0^a^					

^a^Reference category

**Table 4:** FoMO's impact on perceived improvement in sleep habits post-COVID: regression results

Predictor	Categories	Estimate	SE	Wald	*p*	95% confidence interval	
						Lower bound	Upper bound
FOMO		− 0.027	0.013	4.297	**0.038**	− 0.052	− 0.001
Age		− 0.029	0.021	1.834	0.176	− 0.071	0.013
Gender	Male	0.011	0.491	0.000	0.983	− 0.952	0.973
	Female	0^a^					
Sexual orientation	Heterosexual	− 0.629	0.546	1.328	0.249	− 1.698	0.441
	Homosexual	− 1.024	0.957	1.145	0.285	− 2.901	0.852
	Bi-sexual	− 0.928	0.651	2.029	0.154	− 2.205	0.349
	Other	0^a^					
Ethnicity	Asian	− 0.761	0.861	0.782	0.377	− 2.447	0.926
	Black/African American	− 0.931	0.664	1.968	0.161	− 2.231	0.370
	European	− 0.167	0.739	0.051	0.822	− 1.615	1.282
	Latinx	− 1.699	0.623	7.447	0.006	− 2.920	− 0.479
	Middle Eastern	− 1.458	1.012	2.073	0.150	− 3.442	0.526
	Mixed	− 1.513	0.779	3.775	0.052	− 3.039	0.013
	Other	0^a^					
Education	High school	− 1.630	1.197	1.854	0.173	− 3.977	0.717
	Currently undergraduate student	− 0.468	0.855	0.300	0.584	− 2.144	1.208
	Currently graduate student	0.268	0.867	0.096	0.757	− 1.432	1.969
	Master's Degree	0.602	0.946	0.405	0.524	− 1.252	2.456
	Beyond Masters/PhD/Professional Degree	0a					
Birthplace	Outside of USA	− 0.247	0.381	0.421	0.517	− 0.995	0.500
	USA	0^a^					

^a^Reference category

**Table 5:** FoMO's impact on perceived improvement in physical health post-COVID: regression results

Predictor	Categories	Estimate	SE	Wald	*p*	95% confidence interval	
						Lower bound	Upper bound
**FOMO**		**− 0.023**	**0.013**	**3.125**	**0.077**	**− 0.047**	**0.002**
Age		− 0.040	0.021	3.449	0.063	− 0.081	0.002
Gender	Male	0.502	0.488	1.057	0.304	− 0.454	1.457
	Female	0^a^					
Sexual orientation	Heterosexual	0.341	0.535	0.406	0.524	− 0.708	1.390
	Homosexual	− 1.218	0.953	1.634	0.201	− 3.085	0.650
	Bi-sexual	0.457	0.639	0.512	0.474	− 0.795	1.709
	Other	0^a^					
Ethnicity	Asian	− 0.753	0.846	0.792	0.373	− 2.411	0.905
	Black/African American	0.187	0.649	0.083	0.773	− 1.085	1.460
	European	− 0.373	0.728	0.263	0.608	− 1.800	1.053
	Latinx	− 0.684	0.600	1.302	0.254	− 1.859	0.491
	Middle Eastern	− 0.621	0.994	0.390	0.532	− 2.569	1.327
	Mixed	− 1.180	0.763	2.391	0.122	− 2.676	0.316
	Other	0^a^					
Education	High school	− 1.409	1.181	1.424	0.233	− 3.724	0.905
	Currently undergraduate student	− 0.253	0.843	0.090	0.764	− 1.904	1.399
	Currently graduate student	0.493	0.855	0.332	0.564	− 1.183	2.169
	Master's Degree	0.938	0.934	1.009	0.315	− 0.892	2.768
	Beyond Masters/PhD/Professional Degree	0^a^					
Birthplace	Outside of USA	− 0.079	0.376	0.044	0.833	− 0.817	0.658
	USA	0^a^					

^a^Reference category

**Table 6:** FoMO's impact on perceived improvement in mental health post-COVID: regression results

Predictor	Categories	Estimate	SE	Wald	*p*	95% Confidence Interval	
						Lower Bound	Upper Bound
**FOMO**		**− 0.048**	**0.013**	**13.354**	**< 0.001**	**− 0.074**	**− 0.022**
Age		− 0.012	0.021	0.321	0.571	− 0.053	0.029
Gender	Male	− 0.149	0.484	0.095	0.758	− 1.097	0.799
	Female	0^a^					
Sexual orientation	Heterosexual	0.922	0.542	2.895	0.089	− 0.140	1.983
	Homosexual	0.690	0.943	0.536	0.464	− 1.158	2.538
	Bi-sexual	1.405	0.649	4.687	0.030	0.133	2.678
	Other	0^a^					
Ethnicity	Asian	− 0.553	0.849	0.425	0.515	− 2.218	1.111
	Black/African American	− 0.825	0.652	1.599	0.206	− 2.103	0.453
	European	0.061	0.728	0.007	0.933	− 1.366	1.489
	Latinx	− 0.495	0.599	0.683	0.409	− 1.670	0.679
	Middle Eastern	− 1.035	1.004	1.062	0.303	− 3.003	0.934
	Mixed	− 0.159	0.760	0.044	0.834	− 1.648	1.330
	Other	0a					
Education	High school	− 0.037	1.172	0.001	0.975	− 2.335	2.260
	Currently undergraduate student	0.787	0.842	0.874	0.350	− 0.863	2.437
	Currently graduate student	1.410	0.860	2.688	0.101	− 0.276	3.096
	Master's Degree	1.763	0.942	3.502	0.061	− 0.084	3.609
	Beyond Masters/PhD/Professional Degree	0^a^					
Birthplace	Outside of USA	− 0.251	0.377	0.442	0.506	− 0.989	0.488
	USA	0^a^					

^a^Reference category

**Table 7:** FoMO's impact on perceived improvement in emotional life post-COVID: regression results

Predictor	Categories	Estimate	SE	Wald	*p*	95% confidence interval	
						Lower bound	Upper bound
**FOMO**		**− 0.053**	**0.013**	**15.695**	**< 0.001**	**− 0.080**	**− 0.027**
Age		− 0.032	0.021	2.246	0.134	− 0.074	0.010
Gender	Male	0.226	0.486	0.217	0.641	− 0.727	1.179
	Female	0^a^					
Sexual orientation	Heterosexual	0.166	0.536	0.096	0.757	− 0.884	1.216
	Homosexual	− 0.353	0.942	0.140	0.708	− 2.199	1.493
	Bi-sexual	0.551	0.641	0.738	0.390	− 0.706	1.807
	Other	0^a^					
Ethnicity	Asian	− 1.425	0.862	2.731	0.098	− 3.115	0.265
	Black/African American	− 1.222	0.668	3.347	0.067	− 2.531	0.087
	European	− 0.078	0.738	0.011	0.916	− 1.524	1.368
	Latinx	− 1.118	0.617	3.286	0.070	− 2.326	0.091
	Middle Eastern	− 1.387	1.011	1.883	0.170	− 3.369	0.594
	Mixed	− 1.030	0.773	1.774	0.183	− 2.546	0.486
	Other	0^a^					
Education	High school	− 0.062	1.175	0.003	0.958	− 2.366	2.242
	Currently undergraduate student	1.038	0.847	1.504	0.220	− 0.621	2.698
	Currently graduate student	1.857	0.871	4.546	0.033	0.150	3.564
	Master's Degree	1.074	0.933	1.325	0.250	− 0.755	2.904
	Beyond Masters/PhD/Professional Degree	0^a^					
Birthplace	Outside of USA	− 0.174	0.379	0.211	0.646	− 0.917	0.569
	USA	0^a^					

^a^Reference category

**Table 8:** FoMO's impact on perceived improvement in social life post-COVID: regression results

Predictor	Categories	Estimate	SE	Wald	*p*	95% confidence interval	
						Lower bound	Upper bound
**FOMO**		**− 0.053**	**0.014**	**14.986**	**< 0.001**	**− 0.079**	**− 0.026**
Age		− 0.036	0.022	2.731	0.098	− 0.078	0.007
Gender	Male	0.724	0.496	2.132	0.144	− 0.248	1.696
	Female	0^a^					
Sexual orientation	Heterosexual	− 0.019	0.541	0.001	0.971	− 1.080	1.041
	Homosexual	− 1.255	0.956	1.726	0.189	− 3.128	0.618
	Bi-sexual	0.530	0.648	0.669	0.413	− 0.740	1.799
	Other	0^a^					
Ethnicity	Asian	− 0.340	0.855	0.158	0.691	− 2.016	1.336
	Black/African American	− 0.319	0.658	0.236	0.627	− 1.608	0.970
	European	− 0.309	0.739	0.175	0.676	− 1.757	1.140
	Latinx	− 0.714	0.608	1.379	0.240	− 1.906	0.478
	Middle Eastern	1.441	1.060	1.847	0.174	− 0.637	3.519
	Mixed	− 0.789	0.771	1.047	0.306	− 2.299	0.722
	Other	0^a^					
Education	High school	− 2.341	1.204	3.779	0.052	− 4.701	0.019
	Currently undergraduate student	− 0.390	0.852	0.209	0.647	− 2.061	1.281
	Currently graduate student	0.503	0.865	0.339	0.561	− 1.192	2.199
	Master's Degree	0.661	0.942	0.492	0.483	− 1.186	2.508
	Beyond Masters/PhD/Professional Degree	0^a^					
Birthplace	Outside of USA	− 1.106	0.393	7.937	0.005	− 1.875	− 0.336
	USA	0^a^					

^a^Reference category

**Table 9:** FoMO's impact on perceived improvement in professional development: regression results

Predictor	Categories	Estimate	SE	Wald	*p*	95% confidence interval	
						Lower bound	Upper bound
**FOMO**		− 0.039	0.013	8.500	0.004	− 0.065	− 0.013
Age		− 0.006	0.021	0.077	0.781	− 0.048	0.036
Gender	Male	0.968	0.500	3.741	0.053	− 0.013	1.949
	Female	0^a^					
Sexual orientation	Heterosexual	0.132	0.542	0.059	0.808	− 0.930	1.194
	Homosexual	0.111	0.957	0.013	0.908	− 1.765	1.987
	Bi-sexual	− 0.077	0.646	0.014	0.906	− 1.342	1.189
	Other	0^a^					
Ethnicity	Asian	− 0.619	0.853	0.527	0.468	− 2.291	1.053
	Black/African American	− 0.188	0.655	0.083	0.774	− 1.472	1.095
	European	0.561	0.738	0.577	0.447	− 0.886	2.008
	Latinx	0.141	0.603	0.055	0.815	− 1.041	1.323
	Middle Eastern	1.164	1.046	1.238	0.266	− 0.886	3.213
	Mixed	0.358	0.769	0.217	0.641	− 1.149	1.865
	Other	0^a^					
Education	High school	0.966	1.190	0.660	0.417	− 1.365	3.298
	Currently undergraduate student	1.475	0.860	2.942	0.086	− 0.210	3.160
	Currently graduate student	2.357	0.885	7.099	0.008	0.623	4.091
	Master's Degree	1.443	0.946	2.324	0.127	− 0.412	3.298
	Beyond Masters/PhD/Professional Degree	0^a^					
Birthplace	Outside of USA	− 0.803	0.388	4.280	0.039	− 1.564	− 0.042
	USA	0^a^					

^a^Reference category

**Table 10:** FoMO's impact on perceived improvement in financial situation: regression results

Predictor	Categories	Estimate	SE	Wald	*p*	95% confidence interval	
						Lower bound	Upper bound
**FOMO**		**− 0.038**	**0.013**	**8.331**	**0.004**	**− 0.064**	**− 0.012**
Age		− 0.022	0.021	1.108	0.293	− 0.064	0.019
Gender	Male	− 0.088	0.485	0.033	0.856	− 1.039	0.863
	Female	0^a^					
Sexual orientation	Heterosexual	0.561	0.536	1.096	0.295	− 0.490	1.612
	Homosexual	0.209	0.945	0.049	0.825	− 1.644	2.062
	Bi-sexual	0.180	0.638	0.080	0.778	− 1.071	1.431
	Other	0^a^					
Ethnicity	Asian	0.560	0.850	0.434	0.510	− 1.106	2.226
	Black/African American	− 0.438	0.650	0.455	0.500	− 1.712	0.835
	European	0.053	0.730	0.005	0.942	− 1.378	1.485
	Latinx	− 0.326	0.598	0.298	0.585	− 1.499	0.846
	Middle Eastern	1.414	1.042	1.841	0.175	− 0.628	3.456
	Mixed	− 0.541	0.760	0.507	0.476	− 2.031	0.949
	Other	0^a^					
Education	High school	− 0.863	1.179	0.536	0.464	− 3.174	1.448
	Currently undergraduate student	− 1.156	0.851	1.846	0.174	− 2.823	0.512
	Currently graduate student	0.297	0.860	0.120	0.729	− 1.388	1.982
	Master's Degree	0.954	0.943	1.023	0.312	− 0.895	2.803
	Beyond Masters/PhD/Professional Degree	0^a^					
Birthplace	Outside of USA	− 0.869	0.385	5.090	0.024	− 1.624	− 0.114
	USA	0^a^					

^a^Reference category
